# White matter connectivity in children with autism spectrum disorders: a tract-based spatial statistics study

**DOI:** 10.1186/1471-2377-12-148

**Published:** 2012-11-29

**Authors:** Lucia Billeci, Sara Calderoni, Michela Tosetti, Marco Catani, Filippo Muratori

**Affiliations:** 1 Institute of Clinical Physiology, National Council of Research (CNR), Pisa, Italy; 2 IRCCS Stella Maris Foundation, Pisa, Italy; 3 NatBrainLab, Institute of Psychiatry, King’s College London, London, UK; 4 Department of Developmental Medicine, University of Pisa, Pisa, Italy

**Keywords:** ASD, TBSS, Tractography, Arcuate fasciculs, Language

## Abstract

**Background:**

Autism spectrum disorders (ASD) are associated with widespread alterations in white matter (WM) integrity. However, while a growing body of studies is shedding light on microstructural WM alterations in high-functioning adolescents and adults with ASD, literature is still lacking in information about whole brain structural connectivity in children and low-functioning patients with ASD. This research aims to investigate WM connectivity in ASD children with and without mental retardation compared to typically developing controls (TD).

**Methods:**

Diffusion tensor imaging (DTI) was performed in 22 young children with ASD (mean age: 5.54 years) and 10 controls (mean age: 5.25 years). Data were analysed both using the tract-based spatial statistics (TBSS) and the tractography. Correlations were investigated between the WM microstructure in the identified altered regions and the productive language level.

**Results:**

The TBSS analysis revealed widespread increase of fractional anisotropy (FA) in major WM pathways. The tractographic approach showed an increased fiber length and FA in the cingulum and in the corpus callosum and an increased mean diffusivity in the indirect segments of the right arcuate and the left cingulum. Mean diffusivity was also correlated with expressive language functioning in the left indirect segments of the arcuate fasciculus.

**Conclusions:**

Our study confirmed the presence of several structural connectivity abnormalities in young ASD children. In particular, the TBSS profile of increased FA that characterized the ASD patients extends to children a finding previously detected in ASD toddlers only. The WM integrity abnormalities detected may be relevant to the pathophysiology of ASD, since the structures involved participate in some core atypical characteristics of the disorder.

## Background

Autistic Disorder, Pervasive Developmental Disorder-Not Otherwise Specified and Asperger Syndrome (referred to hereafter as Autistic Spectrum Disorders, -ASD-) are a group of neurodevelopmental pathologies characterized by a triad of early symptoms, namely social impairments, communication impairments and rigid and repetitive behaviours. There is increasing evidence that the heterogeneity and complexity of core and associated ASD symptoms can be explained as a fundamental impairment in brain connectivity [1-3]. In this perspective, the advent of Diffusion Tensor Imaging (DTI), a recent noninvasive magnetic-resonance-based method specifically sensitive to the presence of subtle white matter (WM) abnormalities, has allowed for a better understanding of ASD neuroanatomical underpinnings. DTI is based on measurement of water diffusion through cellular compartments in vivo [4]. In particular, compared to more isotropic movement of water in grey matter, WM is characterized by anisotropic water diffusion, referable to the anatomical configuration of structured axonal membranes and myelin sheaths [5]. Several measures can be extracted from DTI analyses, among which are fractional anisotropy (FA), and mean diffusivity (MD). FA is considered a valid measure of WM structural integrity, sensitive to anomalies in axonal density, diameter, myelination, interaxonal spacing and coherence of directional alignment of fibers within WM tracts [6-8], while MD represents a directionally independent measure of the average diffusivity that reflects the degree of myelination, interstitial space and axonal density [9]. A schematic distinction of DTI methods of analysis includes: 1. Region-of-interest (ROI) approach and 2. Whole-brain analysis. Whole-brain analysis investigates the entire brain without a priori ROI selection and thus could be of particular relevance to ASD, in which WM tracts implicated in the etiopathogenesis of the disorder are diffuse and not completely clarified. Among whole-brain approaches, a further innovative method of analysis is tract-based spatial statistics (TBSS) [10]. This analysis method provides some advantages with respect to traditional voxel-based analysis (VBA), since it does not require smoothing and it allows for a higher spatial comparability.

In the last decade, several papers based on DTI have been published, difficult to compare with each other, depending on substantial differences not only as to the clinical and demographic characteristics of the samples, but also as to the methods of analysis. Notably, an age-related pattern of structural changes in the ASD brain is a well-known finding [11]. Specifically, the neuroanatomical differences between ASD patients and typical controls appear to be more prominent in younger subjects and to attenuate with increasing age. This issue can partly explain the conflicting results of ASD imaging data. In particular, almost all the DTI studies on ASD adolescents and adults agree on a FA reduction with respect to typical controls.

To date, only few voxel-based DTI reports have been performed to investigate whole brain WM abnormalities in young children with ASD. Ben Bashat and co-workers [12] first examined a young age group (range 1.8-3.3 years) of ASD subjects, revealing FA increase in several brain areas, with particular involvement of the left and anterior regions. Subsequent reports in ASD early childhood age range highlight a picture of concomitant FA decrease and increase, with various anatomic locations [13,14] or of reduced FA only [15]. More recently, a limited number of studies involving young children with ASD have specifically investigated the structural connectivity through the TBSS approach [16,17]. The two above mentioned studies showed contradictory results, explained, at least in part, by differences in IQ as well as in other clinical features of patients. Specifically, they are characterized by reduced FA in right uncinate fasciculus, left arcuate fasciculus, right cingulum and corpus callosum [16] and increased FA in genu and midbody of the corpus callosum, in left superior longitudinal fasciculus and in bilateral cingulum [17].

As far as the correlation between clinical data and DTI indices, few papers have examined the relationship between WM microstructure integrity and language abilities of ASD patients. Impairments in language and communication skills represent a core diagnostic feature of ASD. In fact, ASD expressive language abilities are highly heterogeneous and may range from nearly absent (nonverbal patients) to a fluent speech production. However, deficits in one or more linguistic domains (phonology, syntax, semantics and pragmatics) are always present in these subjects [18]. A previous study found an inverse correlation between FA values and communication impairment ADI-R scores in fronto-striatal areas and posterior corpus callosum [14], whereas others reported no association between measures of structural connectivity and language functioning [13,19].

In this study, the WM integrity of ASD children is investigated by using DTI with TBSS analysis method and tractography. In particular, the purpose of this research is three-fold: first, to examine possible brain region differences in the WM microstructure integrity between ASD children and typically developing controls (TD); second, to analyse tract-specific differences between ASD group and TD; third, to evaluate whether a relationship between productive language skills and indexes of WM abnormalities in language-related pathways is present in ASD subjects.

## Methods

### Subjects

The final sample included 22 ASD patients and a control group composed of 10 typically developing children. ASD sample consisted of 11 children with autistic disorder (AD) and 11 children with pervasive developmental disorder – not otherwise specified (PDD-NOS). The sequence required for DTI analysis was acquired in all ASD patients who sequentially underwent a brain MRI examination between October 2008 and September 2010 and satisfied the following inclusion/exclusion criteria. The main inclusion criteria were: diagnosis of an ASD; age between 2 and 12 years; a non-verbal IQ≥35 (NVIQ). ASD diagnosis was made according to the DSM-IV-TR criteria (APA; 2000) by a multidisciplinary team including a senior child psychiatrist, an experienced clinically trained research child psychologist and a speech-language pathologist during 5–7 days of extensive evaluation. The ASD diagnosis was confirmed by the Autism Diagnostic Observation Schedule-Generic (ADOS-G; [20]) in 17 of 22 patients and supported by an high Childhood Autism Rating Scale (CARS; [21]) score (≥37, range 37–52) in the remaining 5 ASD. Exclusion criteria included: 1) neurological syndromes or focal neurological signs; 2) dysmorphic features suggestive of a genetic syndrome; 3) significant sensory impairment (*e.g.*, blindness, deafness); 4) anthropometric parameters (weight, height and head circumference) lying outside two SD from the mean of normal subjects; 5) anamnesis of birth asphyxia, premature birth, head injury or epilepsy; 6) use of any psychotropic medication; 7) presence or history of any other axis I mental disorder*.* Moreover, after MRI/DTI acquisition, six patients were excluded due to 8) anomalies detected by MRI (one patient); 9) insufficient image quality (four patients); 10) for children under 48 months, lack of follow-up after 48 months of chronological age confirming the clinical diagnosis of ASD (one patient). This latter exclusion criterion is motivated by the possible instability of ASD diagnosis at very young ages.

ASD patients performed also the recommended laboratory tests to rule-out medical causes of ASD, including audiometry, thyroid hormone disorders, high-resolution karyotyping, DNA analysis of FRA-X and screening tests for inborn errors of metabolism (plasma and urine aminoacid analysis, urine organic acid measurement, urine mucopolysaccarides quantitation, plasma and urine creatine and guanidinoacetate analysis).

The TD subjects were recruited among patients who underwent a brain MRI exam ordered by a physician for a variety of clinical reasons such as headache, head trauma, cataract, dizziness. Inclusion criteria were: 1) a standardized evaluation of cognitive abilities 2) clinical data records providing sufficient information to ensure the lack of neurological, behavioural or developmental disorders. The control group was selected so as to meet the same exclusionary criteria as the ASD (except the tenth criterion specified above) with the further requirement of no family history of ASD.

### Cognitive and linguistic assessment

A number of well-standardized tests were used to assess intellectual abilities due to differences in the age, verbal skills and functioning level of children. These included: the *Leiter International Performance Scale - Revised*[22], the *Griffiths Mental Development Scale*[23] the Italian version of *Wechsler Preschool and Primary Scale of Intelligence* (WPPSI, [24]) and *Wechsler Intelligence Scales for Children – Revised* (WISC-R, [25]). When the tool provides a mental age (MA), IQ was estimated dividing MA by the child’s chronological age (CA): ([MA/CA] X 100). For this study we consider the non-verbal IQ scores (NVIQ).

The demographic and clinical characteristics of patients and controls are reported in Table 1.

**Table 1:** Participants characteristics

	**Subject groups**		**Significance**
	**Autism spectrum disorder (n=22)**	**Controls (n=10)**	**p value**
Age, years:	5.54 (2.03) 2.88-11.33	5.25 (2.46) 2–11.22	.73
Mean (s.d) range			
IQ (non verbal)	70.54 (23.31) 35-108	98.90 (8.45) 85-108	<.001
Mean (s.d) range			

With regard to expressive language level, according to an authoritative consensus group about productive language abilities in ASD children [26], patients and controls were directly assessed and assigned to one of the following conditions: 1- preverbal communication; 2- first words; 3- word combinations; 4- sentences; 5- complex language.

The percentage of these conditions for both ASD subjects and controls are summarized in Table 2.

**Table 2:** Percentages of language conditions

	**Subject groups**	
	**Autism spectrum disorder (n=22)**	**Controls (n=10)**
Preverbal communication	13.6%	-
First words	31.8%	-
Word combinations	18.2%	10%
Sentences	22.7%	-
Complex language	13.6%	90%

### Image acquisition

Structural and diffusion tensor MRI of the brain were performed on a 1.5 T MR system (Signa Horizon LX, GE Medical System). An axial three-dimensional fast spoiled gradient (fSPGR) dataset covering the whole head was acquired. The parameters were: TR = 12.3 ms, TE = 2.4 ms, voxel resolution 256 x 256, field of view 280 mm, 124 slices, 1.1 mm slice thickness. For the DTI analysis, a multislice echo-planar imaging (EPI) acquisition sequence, using 25 directions of diffusion gradients, was used. After an interpolation automatically applied by the MR system the resolution was 0.7422mm x 0.7422mm x 3mm with a field of view 190mm x 190mm and coverage of the whole brain (TE = 107 ms, TR = 11000 ms, b-value = 1000 s/mm). Both ASD and TD children were sedated with a general anaesthesia with a halogenated agent while spontaneously breathing. The written informed consent from a parent or guardian of children was obtained. The research protocol was approved by the Institutional Review Board of the Clinical Research Institute for Child and Adolescent Neurology and Psychiatry.

### DTI processing

#### Image analysis

White matter microstructure integrity were compared between ASD patients and TD controls by using two methods of analysis: whole brain voxel-based comparison, carried out with tract-based spatial statistics (TBSS [10]) and tractography analysis, built from the TBSS findings.

#### Maps reconstruction

Images were processed using the FSL (FMRIB Software Library, FMRIB, Oxford, UK) [27] software package. For each subject, all images including diffusion weighted and b0 images were corrected for eddy current induced distortion and subject motion effect using FDT (FMRIBs Diffusion Toolbox) [28]. Brain mask was created from the first b0 image using BET (Brain Extraction Tool) [29] and FDT was used to fit the tensor model and to compute the FA, MD, axial diffusivity and radial diffusivity maps.

#### TBSS analysis

Voxelwise analysis was performed using TBSS [10]. First the most representative FA image was identified and all subjects’ FA data were aligned to this target image using the nonlinear registration tool FNIRT [30,31], which uses a b-spline representation of the registration warp field [32]. Next, the mean FA image was created and thinned to create a mean FA skeleton, which represents the centres of all tracts common to the group. A threshold of FA > 0.25 was applied to the skeleton to include only major fiber bundles. Each subject’s aligned FA data was then projected onto this skeleton and the resulting data fed into voxelwise cross-subject statistics. Moreover, for each subject the mean FA for the whole-brain WM skeleton was computed.

#### Statistical analysis

Statistical analysis was performed voxel by voxel to detect regions of significant differences of FA between the two groups of subject. The correlation of FA with age was also investigated introducing this parameter as covariate in the contrast matrix. Individual FA maps were included in a non-parametric permutation-based group model using “randomize” in FSL [33]. The TFCE (Threshold-Free Cluster Enhancement) option in randomize was used in order to avoid the need for the arbitrary initial cluster-forming threshold. Both contrasts were computed using 5000 permutations. Results are reported at corrected threshold p < .03. Univariate analysis of covariance (ANCOVA) was applied in SPSS software (SPSS Inc, Chicago, Ill) in order to compare ASD and controls for differences on mean FA for the whole-brain WM skeleton using age as covariate.

### Tractography

#### Tract reconstruction

**Figure 1:**
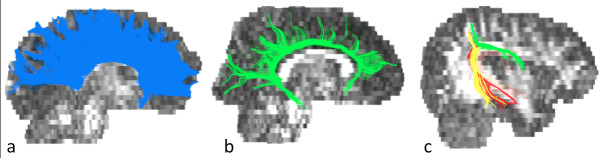
**Selected tracts reconstruction. a**) corpus callosum, **b**) cingulum and **c**) arcuate fasciculus.

Diffusion tensor vectors were computed using ExploreDTI software (http://www.exploredti.com/). After the application of motion and distortion correction, a deterministic tracking algorithm was applied. Tract data were then transformed in NifTi format using a custom MATLAB script including also information on length, FA and MD of each tract. NifTi files were finally imported in TrackVis software (http://www.trackvis.org/) for the reconstruction of the tracks of interest. WM areas that TBSS analysis showed to be significantly different in the two groups were selected for the analysis. We decided to consider only the tracts known to be involved in the core deficits of ASD. The following tracts were reconstructed: the corpus callosum, the cingulum and the arcuate fasciculus (Figure 1). The dissection of these tracts was performed according to the procedure described by Catani and colleagues [34].

The reconstruction of the tracts was performed with an FA threshold of 0.2 to avoid false positives due to artefacts.

#### Tractography outcome measures

For each tract selected for the analysis the following measures were extracted: number of streamlines, mean length of streamlines, volume of the tract, FA, MD, parallel diffusivity and perpendicular diffusivity.

#### Statistical analysis

Statistical comparisons of the tractography outcome measures were performed using SPSS software (SPSS Inc, Chicago, Ill). General linear model (GLM) analysis for repeated measures was used with side (left and right hemisphere) and tracts (cingulum, corpus callosum and the three segments of the arcuate fasciculus) as the within-subject factors and group as between-subjects factor. Then, in order to compare results for the two groups univariate ANOVA was performed on all the tractography measures. No multiple comparison correction was performed [35-37]. The analyses were also repeated using age as covariate in an ANCOVA test. Correlation between the tractography outcome measures and age, NVIQ and language was also investigated using Pearson's correlation coefficient analysis. Correlation with age and NVIQ was investigated both in ASD and controls, while correlation with language was considered only in ASD because all the controls obtained the maximum language score according to their age.

#### Analysis of FA age-dependence

In order to investigate FA age-dependence within the range of age of the subjects, regression scatterplots for whole-brain FA and for FA of the tracts for which significant differences between the two groups were found, each plotted against age, were realized, both for ASD and control children.

## Results

### Group characteristics

There were no significant between-group differences in age (control group mean age 5.54±2.03 years; ASD group mean age 5.25±2.46; p = .73), while, as expected, the NVIQ was found significantly different between the two groups (control group mean NVIQ: 98.90±8.45; ASD group mean NVIQ: 70.54±23.31; p = .0006) (Table 1).

### FA differences between groups

**Figure 2:**
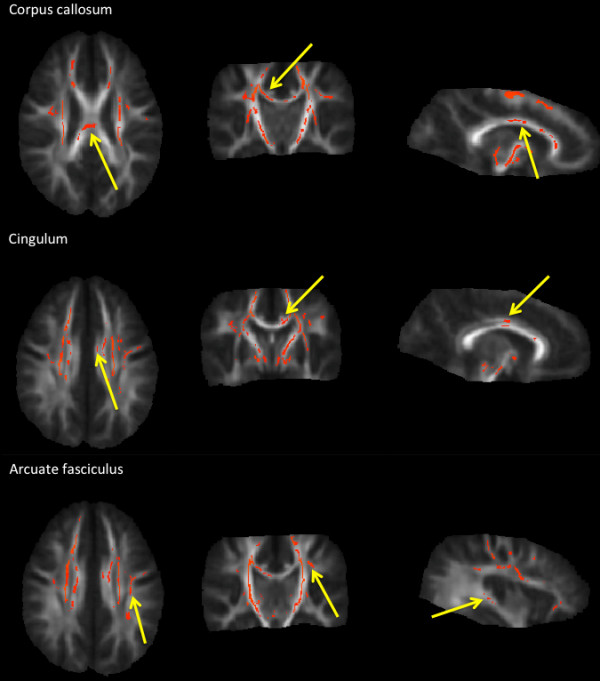
**TBSS.** Results from TBSS analysis of FA maps showing the clusters of significantly increased FA in ASD subjects compared to controls in red (TFCE, p < .03 FWE-corrected). The area selected for the tract analysis are indicated by arrows.

Young children with autism spectrum disorder had a significant increase of FA in a lot of WM areas. In particular, increase in corpus callosum, cingulum, external and internal capsula, arcuate fasciculus was found (p = .03) (Figure 2). No significant correlation with age was found for any of the two groups. No significant difference was found in FA whole-brain WM skeleton between ASD and controls (ASD: mean = 0.41±0.02, controls: mean = 0.40±0.03, p=.41)

### Tract-specific measurements

#### Autism-spectrum disorder vs controls

##### Number length and volume of streamlines

**Figure 3:**
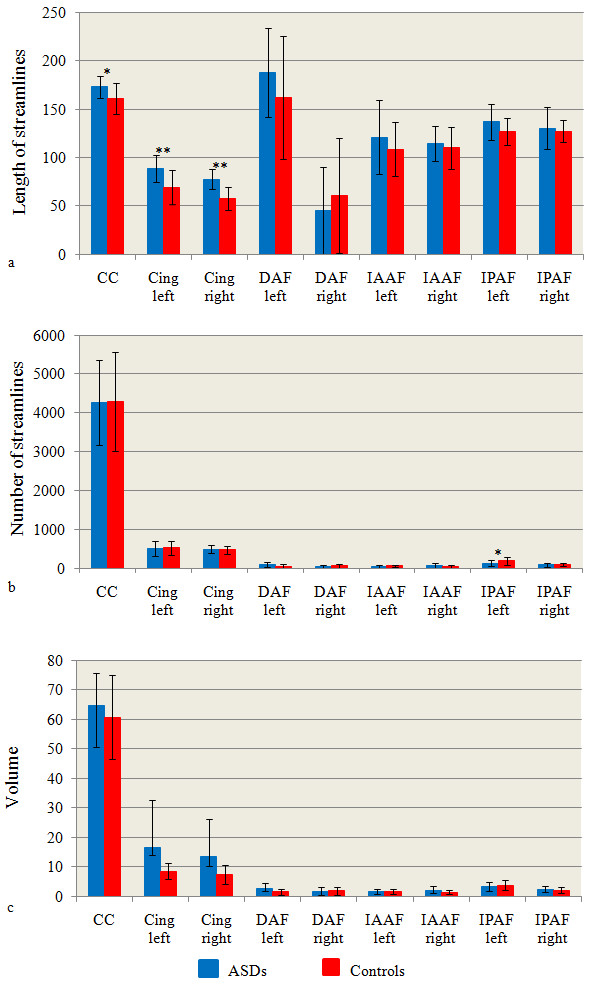
**Histograms of the measures related to streamlines.** Values of number of streamlines (**a**), mean length of streamlines (**b**) and volume (**c**) from all tracts. * p < .05; ** p < .01. ASDs: Autism Spectrum Disorders. CC: corpus callosum; Cing: cingulum; DAF: direct segment of the arcuate fasciculus; IAAF: indirect anterior segment of the arcuate fasciculus; IPAF: indirect posterior segment of the arcuate fasciculus. Units of length are mm and units of volume are mm^3^.

The GLM showed a significant group-by-side-by-tract interaction (p = .01). There was no significant age-by-side (p = .23), age-by-tract (p = .24) or age-by-side-by-tract (p = .06) interaction. Comparison of the individual tracts revealed a significant increase in the length of streamlines bilaterally within the cingulum in ASD group (left cingulum: mean = 88.7±14.2, right cingulum: mean = 77.8±10.4) than in controls (left cingulum: mean = 69.2±17.6; p = .002, right cingulum: mean = 58.2±11.9; p = .0001) and a significant increase in the length of streamlines in the corpus callosum in ASD group (mean = 173.2±11.3) than in controls (mean = 161.1±16.3; p = .02) (Table 3, Figure 3a). There was also a significant reduction in the number of streamlines within the posterior indirect segment of the left arcuate fasciculus in ASD group (mean = 129.5±77.4) than in controls (mean = 190.8±98.5; p = .04) (Table 4, Figure 3b). No significant differences in the volume of the analysed tracts were found (Table 5, Figure 3c). All the analyses remained significant by adding age as covariate.

**Table 3:** Length of streamlines

	**Subject groups**		**Significance**
	**Autism spectrum disorder**	**Controls**	**p value**
CC	173.2 (11.3)	161.1 (16.3)	.02*
Cing left	88.7 (14.2)	69.2 (17.6)	.002**
Cing right	77.8 (10.4)	58.2 (11.9)	<.001**
DAF left	188.1 (46.5)	162.4 (63.5)	.21
DAF right	45.6 (4.05)	60.4 (59.6)	.73
IAAF left	121.2 (38.0)	108.8 (27.9)	.36
IAAF right	114.2 (18.1)	110.2 (21.6)	.59
IPAF left	136.9 (18.9)	126.8 (13.9)	.14
IPAF right	130.4 (21.5)	127.5 (11.6)	.69

Data are expressed as mean (SD). Units of length are mm for mean and standard deviation.

*CC*: corpus callosum; *Cing*: cingulum; *DAF*: direct segment of the arcuate fasciculus; *IAAF*: indirect anterior segment of the arcuate fasciculus; *IPAF*: indirect posterior segment of the arcuate fasciculus.

* Statistically significant at the threshold uncorrected p < .05.

** Significance level at the threshold uncorrected p < .01.

**Table 4:** Number of streamlines

	**Subject groups**		**Significance**
	**Autism spectrum disorder**	**Controls**	**p value**
CC	4270.7 (1088.0)	4298.8 (1270.7)	.95
Cing left	510.3 (192.8)	522.7 (183.7)	.9
Cing right	492.8 (109.7)	470.3 (98.8)	.6
DAF left	90.7 (69.8)	55.3 (46.7)	.17
DAF right	45.6 (45.4)	60.4 (59.6)	.46
IAAF left	43.7 (40.4)	58.4 (34.3)	.32
IAAF right	80.1 (63.8)	46.5 (33.8)	.13
IPAF left	129.5 (77.4)	190.8 (98.5)	.04*
IPAF right	88.7 (45.3)	91.5 (46.1)	.87

Data are expressed as mean (SD).

*CC*: corpus callosum; *Cing*: cingulum; *DAF*: direct segment of the arcuate fasciculus; *IAAF*: indirect anterior segment of the arcuate fasciculus; *IPAF*: indirect posterior segment of the arcuate fasciculus.

* Statistically significant at the threshold uncorrected p < .05.

**Table 5:** Volume

	**Subject groups**		**Significance**
	**Autism spectrum disorder**	**Controls**	**p value**
CC	64.7 (11.1)	60.7 (14.2)	.39
Cing left	16.7 (19.5)	8.6 (2.6)	.2
Cing right	13.4 (18.6)	7.5 (3.1)	.33
DAF left	2.7 (1.8)	1.6 (1.0)	.07
DAF right	1.8 (1.3)	1.9 (1.4)	.88
IAAF left	1.6 (.9)	1.6 (.8)	.88
IAAF right	2.1 (1.3)	1.5 (.8)	.14
IPAF left	3.4 (1.4)	3.9 (1.7)	.35
IPAF right	2.5 (.9)	2.2 (.9)	.47

Data are expressed as mean (SD). Units of length are mm3 for mean and standard deviation.

*CC*: corpus callosum; *Cing*: cingulum; *DAF*: direct segment of the arcuate fasciculus; *IAAF*: indirect anterior segment of the arcuate fasciculus; *IPAF*: indirect posterior segment of the arcuate fasciculus.

#### FA, MD, parallel and perpendicular diffusivity

**Figure 4:**
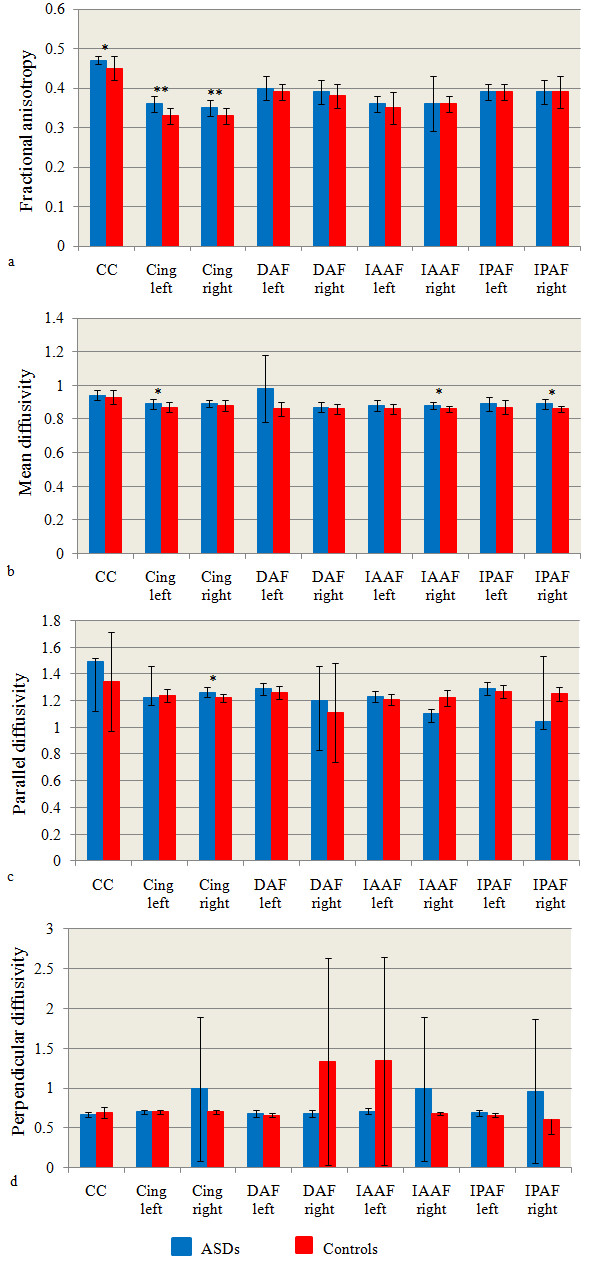
**Histograms of the invariant indices.** Values of fractional anisotropy (FA-**a**), mean diffusivity (MD-**b**) parallel diffusivity (**c**) and perpendicular diffusivity (**d**) from all tracts. * p< .05; ** p < .01. ASDs: Autism Spectrum Disorders. CC: corpus callosum; Cing: cingulum; DAF: direct segment of the arcuate fasciculus; IAAF: indirect anterior segment of the arcuate fasciculus; IPAF: indirect posterior segment of the arcuate fasciculus. Units of MD, parallel diffusivity and perpendicular diffusivity are 10^-3^ mm^2^/s.

FA in ASD group was significantly increased bilaterally in the cingulum (left cingulum: p = .002, right cingulum: p = .003) and in the corpus callosum (p = .01) (Table 6, Figure 4a). There was also an increase of MD in ASD group in comparison to controls in the following areas: within the left cingulum (p = .04), within the right indirect posterior (p = .03) and the right indirect anterior (p = .04) segments of the fasciculus arcuate (Table 7, Figure 4b). Finally, a significant increase in parallel diffusivity in the ASD group was found in the right cingulum (p = .02) (Table 8, Figure 4c). No significant differences in the mean perpendicular diffusivity of the analysed tracts were found (Table 9, Figure 4d). All the analyses remained significant by adding age as covariate

**Table 6:** Fractional anisotropy (FA)

	**Subject groups**		**Significance**
	**Autism spectrum disorder**	**Controls**	**p value**
CC	.47 (.01)	.45 (.03)	.01*
Cing left	.36 (.02)	.33 (.02)	.002**
Cing right	.35 (.02)	.33 (.02)	.003**
DAF left	.4 (.03)	.39 (.02)	.5
DAF right	.39 (.03)	.38 (.03)	.66
IAAF left	.36 (.02)	.35 (.04)	.55
IAAF right	.36 (.07)	.36 (.02)	.78
IPAF left	.39 (.02)	.39 (.02)	.76
IPAF right	.39 (.03)	.39 (.04)	.98

Data are expressed as mean (SD).

*CC*: corpus callosum; *Cing*: cingulum; *DAF*: direct segment of the arcuate fasciculus; *IAAF*: indirect anterior segment of the arcuate fasciculus; *IPAF*: indirect posterior segment of the arcuate fasciculus.

* Statistically significant at the threshold uncorrected p < .05.

** Significance level at the threshold uncorrected p < .01.

**Table 7:** Mean diffusivity (MD)

	**Subject groups**		**Significance**
	**Autism spectrum disorder**	**Controls**	**p value**
CC	.94 (.03)	.93 (.04)	.36
Cing left	.89 (.03)	.87 (.03)	.04*
Cing right	.89 (.02)	.88 (.03)	.58
DAF left	.89 (.02)	.86 (.04)	.38
DAF right	.89 (.03)	.86 (.03)	.15
IAAF left	.89 (.03)	.86 (.03)	.08
IAAF right	.89 (.03)	.86 (.04)	.04*
IPAF left	.89 (.04)	.87 (.04)	.08
IPAF right	.89 (.03)	.86 (.02)	.03*

Data are expressed as mean (SD). Units of MD are 10^-3^ mm^2^/s for mean and standard deviation.

*CC*: corpus callosum; *Cing*: cingulum; *DAF*: direct segment of the arcuate fasciculus; *IAAF*: indirect anterior segment of the arcuate fasciculus; *IPAF*: indirect posterior segment of the arcuate fasciculus.

* Statistically significant at the threshold uncorrected p < .05.

**Table 8:** Parallel diffusivity (D || )

	**Subject groups**		**Significance**
	**Autism spectrum disorder**	**Controls**	**p value**
CC	1.49 (.03)	1.34 (.37)	.06
Cing left	1.22 (.24)	1.24 (.05)	.08
Cing right	1.26 (.04)	1.22 (.03)	.02*
DAF left	1.29 (.04)	1.26 (.05)	.15
DAF right	1.2 (.26)	1.11 (.37)	.49
IAAF left	1.23 (.04)	1.21 (.04)	.17
IAAF right	1.1 (.04)	1.22 (.06)	.38
IPAF left	1.29 (.05)	1.27 (.05)	.13
IPAF right	1.04 (.49)	1.25 8.05)	.18

Data are expressed as mean (SD). Units of D || are 10^-3^ mm^2^/s for mean and standard deviation.

*CC*: corpus callosum; *Cing*: cingulum; *DAF*: direct segment of the arcuate fasciculus; *IAAF*: indirect anterior segment of the arcuate fasciculus; *IPAF*: indirect posterior segment of the arcuate fasciculus.

* Statistically significant at the threshold uncorrected p < .05.

**Table 9:** Perpendicular diffusivity (D ⊥)

	**Subject groups**		**Significance**
	**Autism spectrum disorder**	**Controls**	**p value**
CC	.67 (.03)	.69 (.07)	.14
Cing left	.70 (.03)	.70 (.03)	.77
Cing right	.99 (0.90)	.70 (.03)	.49
DAF left	.68 (.04)	.66 (.03)	.31
DAF right	.68 (.04)	1.33 (1.3)	.16
IAAF left	.71 (.04)	1.34 (1.3)	.15
IAAF right	.99 (0.90)	.68 (.02)	.49
IPAF left	.69 (.04)	.66 (.03)	.09
IPAF right	.96 (0.9)	.61 (.19)	.40

Data are expressed as mean (SD). Units of D ⊥ are 10^-3^ mm^2^/s for mean and standard deviation.

*CC*: corpus callosum; *Cing*: cingulum; *DAF*: direct segment of the arcuate fasciculus; *IAAF*: indirect anterior segment of the arcuate fasciculus; *IPAF*: indirect posterior segment of the arcuate fasciculus.

#### Analysis of FA age-dependence

**Figure 5:**
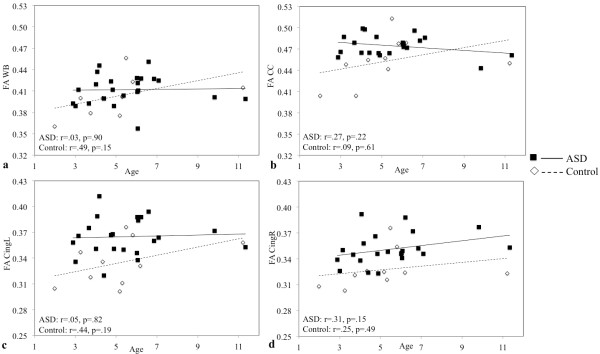
**Scatterplots of FA values against age.** Values of FA against age for whole-brain white matter skeleton (**a**), corpus callosum (**b**), left cingulum (**c**) and right cingulum (**d**). ASD: Autism Spectrum Disorder; WB: whole-brain; CC: corpus callosum; Cing: cingulum.

Linear regression scatterplots were obtained for whole-brain FA, for mean FA of right, and left cingulum and for mean FA of the corpus callosum (Figure 5). The plots show the different FA trajectories and values for ASD and controls. While there is a switch in the trend of FA for whole-brain and corpus callosum at about 7–8 years (first increased in ASD and after increased in controls), for left and right cingulum FA is constantly increased in ASD subjects.

#### Correlations

##### Correlation with age

In control subjects MD was negatively correlated with age in the corpus callosum (r = .69; p = .03), in the right cingulum (r = −.72; p = .02), bilaterally within the direct segment (left: r = −.96; p < .001; right r = −.71; p = .03), bilaterally within the posterior indirect segment (left: r = .85; p = .002; right: r = −.67; p = .03) and in the left anterior indirect segment of the arcuate fasciculus (r = −.88; p = .001). Parallel diffusivity was negatively correlated with age in the right cingulum (r = −.65; p = .04), in the left direct segment (r = .90; p = .002) and in the left posterior indirect segment of the arcuate fasciculus (r = .87; p = .001). Finally perpendicular diffusivity was negatively correlated with age bilaterally within the cingulum (left: r = −.66; p = .04; right r = −.73; p = .02), in the left direct segment (r = .78; p = .02) and in the left posterior indirect segment of the arcuate fasciculus (r = .71; p = .02).

In ASDs there was a positive correlation with age with the number of streamlines (r = .47; p = .02) and the volume (r = .46; p = .03) of the right direct segment of the arcuate fasciculus and a negative correlation with the number of streamlines (r = −.57; p = .005) and the volume of the direct right segment ( r = .46, p = .03) and the indirect posterior right segment of the arcuate fasciculus (r = −.49; p = .02) (Table 10).

**Table 10:** Correlation between age and tract-specific measurements

	**Number of streamlines**				**Length of streamlines**				**Volume**			
	**ASD**		**Controls**		**ASD**		**Controls**		**ASD**		**Controls**	
	**r value**	**p value**	**r value**	**p value**	**r value**	**p value**	**r value**	**p value**	**r value**	**p value**	**r value**	**p value**
CC	-.07	.77	.07	.47	.05	.82	.22	.54	-.14	.53	.31	.39
Cing left	-.13	.30	-.22	.54	.09	.68	-.07	.85	-.18	.43	-.16	.66
Cing right	-.05	.82	-.09	.80	.11	.61	.11	.76	-.15	.50	-.55	.09
DAF left	.25	.25	.39	.29	.30	.16	-.05	.94	.25	.25	.38	.31
DAF right	.47	.02*	.41	.24	.15	.48	-.08	.82	.46	.03*	.39	.26
IAAF left	.23	.29	.17	.64	.01	.02	.46	.18	.15	.49	.39	.27
IAAF right	.41	.05	-.07	.84	.95	.94	-.21	.56	.32	.14	.18	.62
IPAF left	.28	.19	.07	.89	-.15	.49	.61	.06	.12	.59	.20	.57
IPAF right	-.57	.005**	-.28	.44	-.29	.17	.29	.40	-.49	.02*	.005	.99
	**Fractional anisotropy**				**Mean diffusivity**							
	**ASD**		**Controls**		**ASD**		**Controls**					
	**r value**	**p value**	**r value**	**p value**	**r value**	**p value**	**r value**	**p value**				
CC	-.27	.22	.37	.28	-.12	.58	-.69	.03*				
Cing left	.05	.82	.44	.19	-.05	.81	-.47	.17				
Cing right	.31	.15	.25	.49	-.15	.49	-.72	.02*				
DAF left	.21	.35	.33	.43	.08	.73	-.96	<.001**				
DAF right	.008	.97	.003	.99	-.27	.27	-.71	.03*				
IAAF left	.04	.85	.57	.08	-.36	.09	-.88	.001*				
IAAF right	.14	.52	-.22	.54	-.25	.25	-.60	.07				
IPAF left	.22	.31	.18	.62	-.39	.06	-.85	.002**				
IPAF right	-.24	.26	-.25	.49	-.35	.09	-.67	.03*				
	**Parallel diffusivity**				**Perpendicular diffusivity**							
	**ASD**		**Controls**		**ASD**		**Controls**					
	**r value**	**p value**	**r value**	**p value**	**r value**	**p value**	**r value**	**p value**				
CC	-.32	.14	-.10	.78	.07	.77	-.36	.30				
Cing left	.13	.56	-.38	.28	-.06	.79	-.66	.04*				
Cing right	.09	.69	-.65	.04*	.07	.75	-.73	.02*				
DAF left	-.07	.77	-.90	.002*	-.31	.16	-.78	.02*				
DAF right	.12	.63	-.14	.73	-.19	.42	-.01	.98				
IAAF left	-.03	.17	-.33	.35	-.31	.16	-.23	.52				
IAAF right	-.05	.82	-.55	.09	-.20	.37	-.58	.08				
IPAF left	-.37	.12	-.87	.001**	-.38	.07	-.71	.02*				
IPAF right	-.03	.88	-.58	.08	-.08	.72	-.09	.79				

*ASD*: Autism Spectrum Disorder; *CC*: corpus callosum; *Cing*: cingulum; *DAF*: direct segment of the arcuate fasciculus; *IAAF*: indirect anterior segment of the arcuate fasciculus; *IPAF*: indirect posterior segment of the arcuate fasciculus. r: Pearson’s correlation coefficient.

* Statistically significant at the threshold uncorrected p < .05.

** Significance level at the threshold uncorrected p < .01.

#### Correlation with NVIQ

In controls the mean length of streamlines in the left direct segment of the arcuate fasciculus (r = .96; p < .001) and the parallel diffusivity of the left cingulum (r = .66; p = .04) were positively correlated with NVIQ.

No significant correlation with NVIQ was found in ASD subjects (Table 11).

**Table 11:** Correlation between IQ and tract-specific measurements

	**Number of streamlines**				**Length of streamlines**				**Volume**			
	**ASD**		**Controls**		**ASD**		**Controls**		**ASD**		**Controls**	
	**r value**	**p value**	**r value**	**p value**	**r value**	**p value**	**Statistic**	**p value**	**r value**	**p value**	**r value**	**p value**
CC	-.005	.98	.26	.47	-.06	.79	.10	.77	.13	.56	.04	.91
Cing left	.013	.95	-.22	.54	-.13	.55	.33	.34	.22	.31	.34	.27
Cing right	.15	.49	-.09	.80	-.12	.61	.34	.34	.18	.42	.17	.63
DAF left	-.17	.44	.39	.29	-.03	.88	.96	< .001**	-.06	.79	.56	.12
DAF right	-.35	.10	.41	.24	-.05	.82	-.17	.84	-.27	.19	-.14	.69
IAAF left	.07	.75	.17	.64	.26	.23	-.08	.82	.19	.39	-.34	.34
IAAF right	.10	.65	-.07	.84	.26	.24	.06	.87	.23	.30	.15	.68
IPAF left	-.15	.50	.05	.89	.29	.17	.19	.56	-.05	.81	.52	.12
IPAF right	.09	.66	-.28	.44	-.002	.99	.15	.69	.20	.35	-.03	.94
	**Fractional anisotropy**				**Mean diffusivity**							
	**ASD**		**Controls**		**ASD**		**Controls**					
	**r value**	**p value**	**r value**	**p value**	**r value**	**p value**	**r value**	**p value**				
CC	.03	.89	-.24	.51	-.18	.42	.04	.92				
Cing left	-.23	.31	.38	.28	.10	.66	.02	.94				
Cing right	-.20	.37	.07	.84	.33	.13	-.15	.68				
DAF left	-.06	.83	.05	.89	-.36	.10	.06	.83				
DAF right	-.05	.83	.06	.88	-.01	.98	.20	.60				
IAAF left	.07	.75	-.39	.26	-.02	.91	-.81	.82				
IAAF right	.10	.65	-.02	.95	-.01	.98	.43	.21				
IPAF left	-.18	.41	-.09	.79	.10	.63	-.25	.48				
IPAF right	.13	.55	-.08	.82	-.02	.92	.06	.87				
	**Parallel diffusivity**				**Perpendicular diffusivity**							
	**ASD**		**Controls**		**ASD**		**Controls**					
	**r value**	**p value**	**r value**	**p value**	**r value**	**p value**	**r value**	**p value**				
CC	-.13	.56	-.17	.63	-.12	.59	.23	.52				
Cing left	-.14	.54	.66	.04*	.19	.41	-.29	.42				
Cing right	.19	.39	-.05	.89	-.18	.43	-.27	.44				
DAF left	-.03	.89	.07	.84	.07	.75	.07	.84				
DAF right	-.18	.47	-.13	.73	.05	.83	.19	.63				
IAAF left	-.06	.79	-.39	.27	-.05	.82	-.17	.64				
IAAF right	-.27	.23	.42	.23	-.34	.12	.33	.35				
IPAF left	-.05	.81	-.20	.58	.16	.47	-.22	.55				
IPAF right	-.34	.11	.001	.99	.14	.51	-.14	.69				

*ASD*: Autism Spectrum Disorder; *CC*: corpus callosum; *Cing*: cingulum; *DAF*: direct segment of the arcuate fasciculus; *IAAF*: indirect anterior segment of the arcuate fasciculus; *IPAF*: indirect posterior segment of the arcuate fasciculus. r: Pearson’s correlation coefficient.

* Statistically significant at the threshold uncorrected p < .05.

** Significance level at the threshold uncorrected p < .01.

#### Correlation with language

In ASD subjects language abilities were positively correlated bilaterally with the length of streamlines in the indirect anterior segment of the arcuate fasciculus (left: r = .55; p = .006; right: r = .81; p = .009) and negatively correlated with MD (r = −.48; p = .02), and parallel diffusivity (r = −.43; p = .04) of the left indirect anterior segment of the arcuate fasciculus and with MD (r= −.58, p = .005), parallel diffusivity (r= −.58, p = .005) and perpendicular diffusivity (r= −.46, p = .03) of the corpus callosum (Table 12).

**Table 12:** Correlation between language and tract-specific measurements

	**Number of streamlines**		**Length of streamlines**		**Volume**	
	**r value**	**p value**	**r value**	**p value**	**r value**	**p value**
CC	.25	.25	-.06	.79	.24	.28
Cing left	-.11	.63	-.09	.67	.25	.25
Cing right	.24	.27	.14	.53	.21	.35
DAF left	.18	.41	.08	.70	.18	.42
DAF right	-.17	.45	-.08	.71	-.08	.71
IAAF left	-.06	.79	.55	.006**	.15	.48
IAAF right	.11	.63	.55	.009**	.28	.21
IPAF left	.03	.89	.14	.51	.05	.82
IPAF right	-.05	.80	-.29	.18	-.10	.64
	**Fractional anisotropy**		**Mean diffusivity**			
	**r value**	**p value**	**r value**	**p value**		
CC	.14	.53	-.58	.005**		
Cing left	-.05	.83	-.20	.36		
Cing right	.16	.47	-.03	.89		
DAF left	-.04	.87	.26	.25		
DAF right	-.002	.99	-.28	.25		
IAAF left	.18	.42	-.48	.02*		
IAAF right	-.18	.42	-.30	.17		
IPAF left	.07	.75	-.29	.18		
IPAF right	.18	.41	-.39	.07		
	**Parallel diffusivity**		**Perpendicular diffusivity**			
	**r value**	**p value**	**r value**	**p value**		
CC	-.58	.005**	-.46	.03*		
Cing left	.29	.19	-.16	.47		
Cing right	.12	.60	.34	.12		
DAF left	-.38	.08	-.21	.34		
DAF right	.25	.29	-.17	.48		
IAAF left	-.41	.06	-.43	.04*		
IAAF right	.05	.82	-.33	.13		
IPAF left	-.27	.20	-.26	.22		
IPAF right	-.07	.74	.17	.43		

*ASD*: Autism Spectrum Disorder; *CC*: corpus callosum; *Cing*: cingulum; *DAF*: direct segment of the arcuate fasciculus; *IAAF*: indirect anterior segment of the arcuate fasciculus; *IPAF*: indirect posterior segment of the arcuate fasciculus. r: Pearson’s correlation coefficient.

* Statistically significant at the threshold uncorrected p < .05.

** Significance level at the threshold uncorrected p < .01.

## Discussion

In this study, WM integrity in ASD children was investigated following two approaches: the TBSS analysis and the tractography method.

The results provide three key findings: 1) widespread FA increase in WM TBSS of ASD patients, especially evident in the corpus callosum, the cingulum, the arcuate fasciculus (AF) and the internal capsula; 2) significant differences in the corpus callosum, the cingulum and minor differences in the AF, as revealed by the tract analysis; 3) significant correlation between expressive language abilities and tract specific measures of AF, corpus callosum and cingulum.

The first result agrees with previous whole brain DTI works by Ben Bashat and colleagues [12,17], but extends the stage at which FA is increased in ASD patients into an older age. The regression analysis of FA vs. age shows that the whole brain FA increase of our sample was clearly attributable to the subgroup of younger patients, while the older one reported a reduction in FA compared with the TD group. The trends are regionally dependent, since the developmental trend of FA in the corpus callosum crosses atapproximately 7–8 years, while the FA of the cingulum is constantly increased with respect to the control group. It is possible that the corpus callosum, the largest commissural WM tract in the human brain, could influence the global FA and hide the FA trend of other small tracts (e.g. the cingulum).

Most DTI papers on adolescents or adults with ASD show reduced FA in several brain regions, including the corpus callosum [38,39], areas located between the right middle and inferior frontal gyri and in the right centrum semiovale, as well as motor and premotor areas [40] and the superior temporal gyrus [41]. More recently, Shukla and colleagues [42], using the TBSS analysis, found a reduced FA in numerous WM tracts. Moreover, in a study [43] performed on adolescents with ASD, an imbalance of FA was found, as some areas showed an FA increase, while others a FA decrease.

These contradictory results could reflect a change of the trend of FA in young children with respects to adolescents and adults and may be linked to the modification in ASD brain volume, which has an accelerated growth in the first years of life and then shifts to an abnormally slow growth [44-46]. However, the age at which the FA increase is no longer evident varies across studies: Wolff et al. [47] demonstrated in a longitudinal investigation that, by 24 months of age, a decreased FA is evident across all fiber tracts. Conversely, a pattern of FA increase is still present in some WM tracts of children with a mean age of 8.7 years [13], 9.3 years [14], and 13.7 years [43]. In fact, the weak concordance among DTI investigations is largely ascribable to the wide heterogeneity both of ASD clinical phenotype and of methods of analysis, which makes results difficult to compare with each other. Furthermore, experimental artifacts and confounding factors should be taken into account in order to enhance the reliability and comparability of DTI results [48].

Caution is required in interpreting high FA levels, also because this diffusivity change could represent an index of various WM microstructural alterations, like increased myelination, axon size and density, path geometry, and the presence of crossing fiber pathway. To date, the recently developed TBSS analysis has been applied to a limited number of ASD investigations on young autistic children [16,17]. Among the advantages, the TBSS approach offers the possibility to choose as a template the most representative FA map among all the maps of the subjects involved in the study, instead of an external template such as the Talairach or MNI standard spaces. This benefit is particularly important when the study involves young children, since most of the above mentioned standard templates are based on adults.

Second, the reconstruction and the tractographic analysis of the corpus callosum, the cingulum and the AF revealed significant differences in these WM tracts. The corpus callosum is the largest inter-hemispheric tract in the brain and it is thought to be involved in emotional and social functioning, as well as in higher cognitive processes, such as decoding non-literal meaning, affective prosody, and understanding humour [49-51]. Several studies performed on adolescents and adults with ASD reported reduced FA in the corpus callosum [38-40,52,53]. Conversely, an increased FA in the corpus callosum was found when young children with ASD have been investigated [12,17]. In accordance with these latter findings, we confirmed the FA increase in the corpus callosum of young children with ASD. Moreover, a significant increase in the mean length of streamlines in ASD with respect to controls was found.

The cingulate cortex is involved in tasks related to higher-level cognitive processes, like empathic cognition, social behaviour and pain perception in which is more likely to be hypoactivated in ASD subjects [54,55]. Structural abnormalities in the cingulate area have been also demonstrated in DTI studies. Specifically, a FA reduction in the anterior cingulate cortex was found in children as well as in adolescents with ASD [40,52], whilst an increase in the number of streamlines bilaterally within the cingulum was present in early-adults with ASD [56]. More recently, an opposite result was obtained, with a FA increase in the left cingulum of ASD young children [17]. Our results confirmed and extended bilaterally the FA increase within the cingulum. In addition, a significant increase in the length of streamlines of the cingulum in ASD with respect to controls was detected.

The increase in the length of streamlines of both the cingulum and the corpus callosum in the ASD group contrasts with the excessive short-range connectivity hypothesis suggested by several studies [57-59]. On the other hand, it is consistent with two reports of the same research team [16,60], in which the long-range association fibers were significantly longer in the frontal lobe [60], and particularly in the uncinate fasciculus, the AF and the corpus callosum of the ASD sample [16]. While the role of the increased fiber length could be reconductable to a compensatory function with respect to the impaired integration abilities of the ASD brain, the pathogenic mechanism may be ascribed to altered serotonin levels [60]. In fact, several previous studies have indicated that serotonin acts as a neurotrophic factor in axonal outgrowth during development [61,62]. In addition, recent findings point to high serotonin levels in the crucial stages of fetal brain development as a risk-factor for ASD [63].

Conversely, we did not find significant differences in the mean length of streamlines within the AF, although the values are higher in the ASD group with respect to control (Table 3), especially in the left hemisphere.

The AF is a fiber bundle related to language, a function always impaired in ASD subjects, at least from the qualitative point of view. To the best of our knowledge, this is the only study in autism in which the AF is dissected using a three-ROIs approach that allows reconstructing the three tracts of the AF. A previous investigation on ASD adults reported decreased value of FA in the right AF [39], studies on children with ASD described an increased FA and MD bilaterally in the AF [12,17], while another report [16] found an increased MD in the right AF, but a decreased FA in the left AF in ASD group. Our result of an increased MD in the AF is in agreement with the aforementioned study. We found also an increased FA in the TBSS investigation, but not in the tractography analysis, probably referable to the differences between the two techniques. In fact, while the TBSS makes a comparison voxel to voxel on the complete WM skeleton, the tractography is based on the evaluation of the mean value of the invariant indices across each tract: in this way differences in specific areas of the tract might be hidden. Moreover, the tractographic approach can be vexed by the degree of the reproducibility that varies according to WM tract size: while it is possible to obtain a high reproducibility with thick and big tracts (e.g. the corpus callosum), it is harder with thin tracts like the ones that form the AF. The three-ROIs approach for the dissection of the AF allows identifying which specific sub-tracts are compromised in ASD. In particular, we found an increased MD within the indirect segments of the right AF. The MD is a measure directly related to interstitial space between axonal fibers; therefore, the increase in his value could be due to reduced neural or glial cell packing or cell size, as well as decreased water exchange rate between the intra- and extracellular compartments.

The result of increased MD in short associative tracts in ASD subjects with respect to controls is consistent with previously reported DTI studies [38-41,60]. In particular, an analysis of microstructural organization of the AF in ASD patients [19] highlights a significant increase of MD in the ASD left hemisphere.

Conversely, the present study as well as Kumar’s [16] work, localize significant differences in the right hemisphere. However, a comparative discussion between our and previous results [19] is not possible, as differences in the subject selection and tractography methodology are present. First of all, we selected young children and we also included in the sample low functioning and non-verbal individuals with ASD, while the above-mentioned investigators use a high-functioning verbal sample of adolescents. Moreover, these authors only examined the direct segment of the AF, whereas we also considered the indirect segments, in which structural abnormalities (number of streamlines and MD) are particularly prominent.

As far as the correlation between expressive language and tractographic indices, the following findings were found: a) a positive correlation with length of streamlines in the indirect anterior segment of the AF bilaterally and b) a negative correlation with MD and parallel diffusivity of the left indirect anterior segment of the AF. A similar result emerges from a recent study [64], which reveals a significant negative correlation between the MD of AF -considered as a unique tract- and clinical assessment of language function. Furthermore, we identified which are the sub-tracts of the AF related to language impairment.

According to a study of Catani et al. [65], the indirect pathway of the AF appears to relate to semantically-based language functions (e.g. auditory comprehension and vocalization of semantic content), while the direct pathway relates to phonologically-based language functions (e.g. automatic repetition). Considering this assumption, the structural abnormalities in the language pathway in ASD patients could be related to impairment in semantic-based functions. However, this hypothesis should be confirmed with a better characterization of the linguistic profile in the sample subjects. In fact, in the present study, the language score assigned to the subjects arises from an evaluation of the global expressive language ability, without differentiating among the specific domains (i.e. phonology, syntax, semantics and pragmatics). Future research will be addressed to the identification of specific brain-behaviour abnormalities in language-related fiber tracts.

## Conclusions

There are several limitations to our study that should be noted. First, the sample size was relatively small, which might reduce the power of the statistical significance and generalization of the findings. Second, the group of ASD children we have studied reflects the epidemiological characteristics of autistic population both as for the gender distribution (with a skewed for males), that for the level of intellectual functioning (with a frequent mental retardation comorbidity): thus patients and controls were not matched for the sex and the NVIQ. Future investigations, in which separate analyses of data for large subgroups of ASD patients will be conducted (e.g. males and females, high-functioning and low-functioning), are therefore warranted, in order to highlight WM microstructural differences, similarities and distinctive features of each subgroup. Third, subjects included present a wide age range, from 2 to 11 years of age. In order to overcome the latter limitation, (a) we have excluded toddlers under 2 years of age, since changes in DTI indices are age-related [66] and occur, for the most part, within the first 24 months [67]; (b) we have searched for an high age comparability between patients and controls, not only in terms of arithmetic mean, but also of SD and range. Notwithstanding the problematic issues raised above, this study adds to the limited existing literature on young children with ASD. A widespread increased of FA and several tract-specific abnormalities in structures linked to ASD behavioural deficits (e.g. cingulum, corpus callosum and AF) were detected. Moreover, we identified connectivity modifications related to language functioning in the ASD sample. Further studies in which subgroups of ASD subjects will be better characterized according to their language abilities, will allow to identify which structural connectivity abnormalities are related to specific language impairments.

## Abbreviations

ASD: Autism Spectrum Disorder; DTI: Diffusion tensor imaging; WM: White matter; FA: Fractional anisotropy; MD: Mean diffusivity; ROI: Region of interest; TBSS: Tract-based spatial statistics; VBA: Voxel-based analysis; TD: Typically developing; MRI: Magnetic resonance imaging; fSPGR: Fast spoiled gradient recalled; EPI: Echo planar imaging; GLM: General linear model; AF: Arcuate fasciculus.

## Competing interests

The authors declare that they have no competing interests.

## Authors’ contributions

Conceived and designed the experiments: MT/MC/FM. Recruited patients: SC/FM. Collected data and conducted the experiments: LB/SC. Analysed the data: LB. Interpretation of the results: LB/SC/MT/MC/FM. Wrote the paper: LB/SC. All authors read and approved the final manuscript.

## Pre-publication history

The pre-publication history for this paper can be accessed here:

http://www.biomedcentral.com/1471-2377/12/148/prepub
